# Analysis of isolates from Bangladesh highlights multiple ways to carry resistance genes in *Salmonella* Typhi

**DOI:** 10.1186/s12864-019-5916-6

**Published:** 2019-06-28

**Authors:** Nicholas Costa Barroso Lima, Arif M. Tanmoy, Emilie Westeel, Luiz Gonzaga Paula de Almeida, Alain Rajoharison, Maksuda Islam, Hubert P. Endtz, Samir K. Saha, Ana Tereza Ribeiro de Vasconcelos, Florence Komurian-Pradel

**Affiliations:** 1 Departamento de Bioquímica e Biologia Molecular, Universidade Federal do Ceará, Fortaleza, Ceará Brazil; 2 Laboratório Nacional de Computação Científica, Petrópolis, Brazil; 3 Department of Medical Microbiology & Infectious Diseases, Erasmus MC, Rotterdam, the Netherlands; 4 Fondation Mérieux – Laboratoire des Pathogènes Emergents, Lyon, France; 5 Child Health Research Foundation, Department of Microbiology, Dhaka Shishu Hospital, Dhaka, 1207 Bangladesh; 6 Bangladesh Institute of Child Health, Dhaka Shishu Hospital, Dhaka, 1207 Bangladesh

**Keywords:** *Salmonella* Typhi, SGI11, Resistance genes, Typhoid fever, Bangladesh, Comparative genomics

## Abstract

**Background:**

Typhoid fever, caused by *Salmonella* Typhi, follows a fecal-oral transmission route and is a major global public health concern, especially in developing countries like Bangladesh. Increasing emergence of antimicrobial resistance (AMR) is a serious issue; the list of treatments for typhoid fever is ever-decreasing. In addition to IncHI1-type plasmids, *Salmonella* genomic island (SGI) 11 has been reported to carry AMR genes. Although reports suggest a recent reduction in multidrug resistance (MDR) in the Indian subcontinent, the corresponding genomic changes in the background are unknown.

**Results:**

Here, we assembled and annotated complete closed chromosomes and plasmids for 73 *S.* Typhi isolates using short-length Illumina reads. *S.* Typhi had an open pan-genome, and the core genome was smaller than previously reported. Considering AMR genes, we identified five variants of SGI11, including the previously reported reference sequence. Five plasmids were identified, including the new plasmids pK91 and pK43; pK43and pHCM2 were not related to AMR. The pHCM1, pPRJEB21992 and pK91 plasmids carried AMR genes and, along with the SGI11 variants, were responsible for resistance phenotypes. pK91 also contained *qnr* genes, conferred high ciprofloxacin resistance and was related to the H58-sublineage Bdq, which shows the same phenotype. The presence of plasmids (pHCM1 and pK91) and SGI11 were linked to two H58-lineages, Ia and Bd. Loss of plasmids and integration of resistance genes in genomic islands could contribute to the fitness advantage of lineage Ia isolates.

**Conclusions:**

Such events may explain why lineage Ia is globally widespread, while the Bd lineage is locally restricted. Further studies are required to understand how these *S.* Typhi AMR elements spread and generate new variants. Preventive measures such as vaccination programs should also be considered in endemic countries; such initiatives could potentially reduce the spread of AMR.

**Electronic supplementary material:**

The online version of this article (10.1186/s12864-019-5916-6) contains supplementary material, which is available to authorized users.

## Background

Typhoid fever, a major global public health threat, is caused by *Salmonella enterica* serovar Typhi (*S.* Typhi). Due to its fecal-oral transmission route, the disease is most prevalent in the least developed regions of the world in the tropical belt, which also contains the least developed regions of the world. Over 80% of the global burden of 12 million typhoid cases per year occurs in Asia and Africa, mainly among children and adolescents [1–3]. Although the mortality rate is low (1–2%), typhoid fever may lead to long-term physical and mental disabilities if untreated for a long time [4]. Moreover, the huge numbers of typhoid fever cases in developing countries impose a significant economic burden.

Antimicrobial therapy is the most effective treatment for typhoid fever. However, due to increasing levels of antimicrobial resistance (AMR), a small number of cases of treatment failure have been reported, even among patients treated with newer generations of antimicrobials [5–7]. Multidrug resistance (MDR) in *S.* Typhi—defined as co-occurring resistance to ampicillin (amp), chloramphenicol (chl) and cotrimoxazole (sxt)—was first reported in 1973 [8, 9] and resistance to ciprofloxacin (cip) emerged in the early 1990s. The list of available treatment options for typhoid fever has rapidly reduced since the emergence of AMR and treatment regimens have shifted towards quinolones. Extended-spectrum beta-lactams (e.g. ceftriaxone) or macrolides (e.g. azithromycin) are now the most effective treatment options for typhoid fever. However, extended-spectrum beta-lactamase (ESBL)-producing *S.* Typhi have been reported in many countries, and exhibit high levels of resistance to ceftriaxone (cro) [10–12].

In *S.* Typhi, MDR genes are usually carried by an IncHI1-type plasmid [13, 14]. However, a chromosomal *Tn21*-like element has recently been reported as a component of *Salmonella* genomic island 11 (SGI11) [15, 16]. This island has been reported to carry resistance genes for seven different antimicrobial agents, including ampicillin, chloramphenicol, and cotrimoxazole, it integrates into two or more chromosomal locations, and can confer MDR even in the absence of plasmids [16–18]. MDR has also been closely associated with the dominant haplotype H58 (genotype 4.3.1), which exhibits reduced susceptibility to quinolones [16]. Mechanisms of ciprofloxacin resistance (cip-R) usually involve chromosomal point mutations and the acquisition of AMR genes. Such mutations occur in quinolone resistance-determining regions (QRDR), which correspond to multiple locations on the DNA gyrase (*gyrA* and *gyrB*) and topoisomerase IV (*parC* and *parE*) genes [19–22]. The presence of plasmid-mediated quinolone resistance (PMQR) genes, such as *qnr,* and overexpression of efflux pump genes can also contribute to quinolone resistance [23, 24].

Although H58 is still the most prevalent MDR *S.* Typhi variant, a reduction in the frequency of isolation of H58-MDR strains has been reported. These isolates are only resistant to one or two antibiotics, i.e., ampicillin, chloramphenicol, or cotrimoxazole [25–29]. Nonetheless, the genomic changes to chromosomes or plasmids responsible for such H58 non-MDR phenotypes are yet to be described.

In this study, we generated the complete closed chromosome sequences and accessory plasmid sequences for 73 *S.* Typhi strains isolated in Bangladesh between 1999 and 2013; the strains were selected for this study according to their antimicrobial resistance profile. We annotated and studied the core and pan-genomes of all isolates (*n* = 73). The genetic elements responsible for AMR (e.g. genes, mutations, genomic islands) and their locations (plasmids or chromosome) were analyzed and compared with the resistance phenotypes. We also assessed the presence, location and gene contents of SGI11 and plasmids and their associations with resistance phenotypes.

## Results

### General genomic features and comparative genomics

**Fig. 1:**
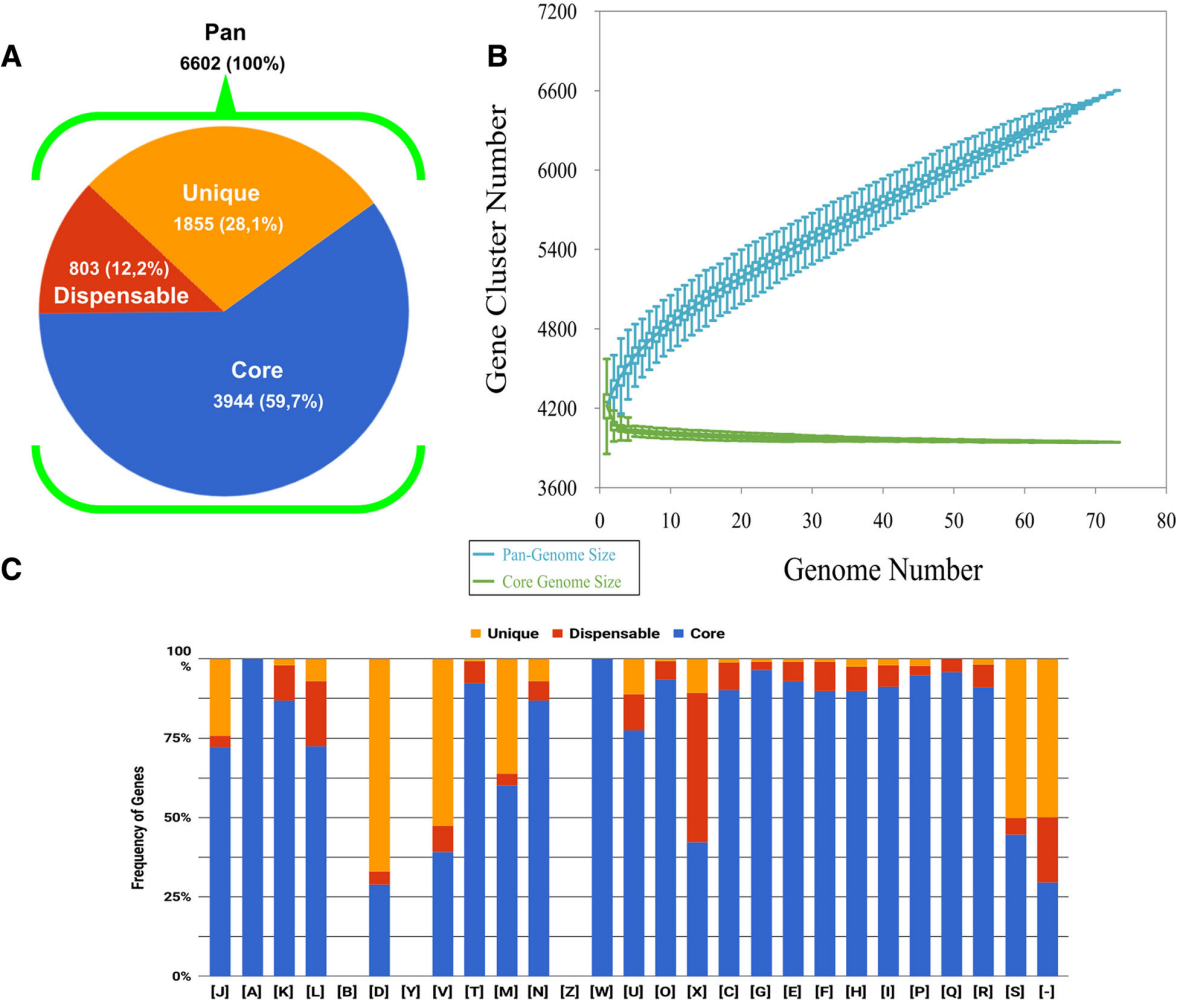
Pan and core genome analysis. **a** The pan-genome of the 73 *Salmonella* Typhi isolates contains 6602 genes. The pan-genome can be further divided into the unique genome (orange), dispensable genome (red) and core genome (blue), depending on how many isolates share a given gene. **b** Core and pan-genome curves. The number of core genes stabilizes after the addition of the tenth genome. The pan-genome is open according to the fitting parameter γ = 0.67. **c** Distribution of genes in the unique, dispensable and core genomes on in each COG class. COG classes are as follows: [J] Translation, ribosomal structure and biogenesis; [A] RNA processing and modification; [K] Transcription; [L] Replication, recombination and repair; [B] Chromatin structure and dynamics; [D] Cell cycle control, cell division, chromosome partitioning; [Y] Nuclear structure; [V] Defense mechanisms; [T] Signal transduction mechanisms; [M] Cell wall/membrane/envelope biogenesis; [N] Cell motility; [Z] Cytoskeleton; [W] Extracellular structures; [U] Intracellular trafficking, secretion, and vesicular transport; [O] Post-translational modification, protein turnover, chaperones; [X] Mobilome: prophages, transposons; [C] Energy production and conversion; [G] Carbohydrate transport and metabolism; [E] Amino acid transport and metabolism; [F] Nucleotide transport and metabolism; [H] Coenzyme transport and metabolism; [I] Lipid transport and metabolism; [P] Inorganic ion transport; [Q] Secondary metabolites biosynthesis, transport and catabolism; [R] General function prediction only; [S] Function unknown; [−] Unclassified

The chromosomes of all 73 *S.* Typhi isolates were assembled and ranged from 4,773,823 to 4,897,593 base pairs (bp) in size (Additional file 1: Table S2). The GC content of all chromosomes was 53%. Automatic gene annotation showed an average of 4236 (median 4230) chromosomal genes, with an average size of 643 bp per coding sequence (Additional file 1: Table S2). The numbers of genes encoding hypothetical proteins, tRNAs, rRNAs, and pseudogenes in all isolates ranged from 871 to 949, 75–84, 21–24, and 171–195, respectively (Additional file 1: Table S2). The lowest and highest ANIb/ANIm values were 99.89–100/99.85–99.92 respectively (Additional file 1: Table S2). The core-genome, defined as the part of the genome common to all isolates, contained 3944 genes, representing 93% of the average gene content of the isolates (Fig. 1a). The dispensable genome (the set of genes shared by some—but not all—isolates) contained 803 genes, while the unique genome (genes present in only one isolate) contained 1855 genes (Fig. 1a). The pan-genome, corresponding to the sum of the core, dispensable and unique genomes was composed of 6602 genes. The curves for the pan and core genomes (Fig. 1b) indicated the number of core genes (green line) stabilized after the addition of the tenth genome. The pan-genome fitting parameter (γ = 0.67; blue line) indicates an open pan-genome [30]. Figure 1c shows the proportion of clusters of orthologous group (COG) classes for the core, dispensable and unique genomes. As the core genome represented 93% of the average number of genes in the isolates, it is reasonable that core genes make the bulk of COG classes (Fig. 1c). Moreover, as typical for bacteria, none of the detected genes were related to nuclear structure (class Y); genes in COG classes B (*Chromatin structure and dynamics*) and Z (*Cytoskeleton*) were not detected either. A number of classes, including K (*Transcription*), L (*Replication, recombination, and repair*), and X (*Mobilome: prophages, transposons*) were represented more frequently in the dispensable genome than the unique and core genomes. We verified the location of the genes in classes K, L and X; 49, 39 and 14% of those genes were within prophage regions. In the unique genome set, the most common gene classes were J (*Translation, ribosomal structure, and biogenesis*), D (*Cell cycle control, cell division, chromosome partitioning*), V (*Defense mechanisms*) and M (*Cell wall/membrane/envelope biogenesis*).

### Presence of *Salmonella* genomic island 11 (SGI11)

**Fig. 2:**
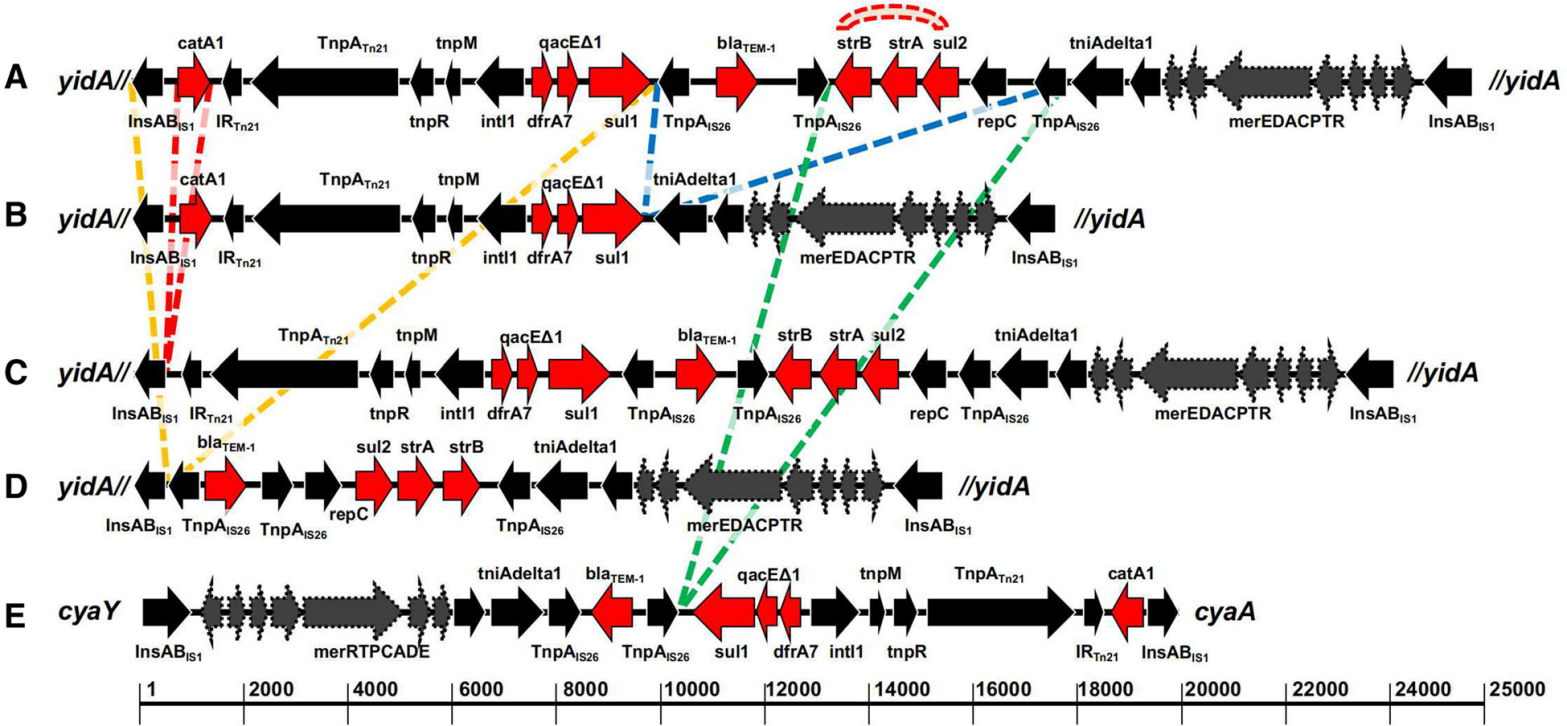
The genomic island SGI11 and its variants. Red arrows are resistance genes, grey dashed arrows are mercury metabolism genes, black arrows are insertion elements and transposases. Colored dashed lines denote segments in the archetypical SGI11 (**a**) that are absent in other variants. Red dashed bridge denotes an inversion. *yidA*// (double forward dash means a truncation) and *cyaY-cyaA* denote the sites of insertion of the islands. **b** SGI11*b*. **c** SGI11*c*. **d** SGI11*d*. **e** SGI11*e*. The ruler gives an approximate island size

Twenty-one of the 73 isolates harbored a genomic island similar to SGI11. Manual curation of gene content revealed that not all of these genomic islands were archetypical. Some isolates had the same gene content as SGI11 (*n* = 8), and we identified four variants that we named SGI11*b* (*n* = 9), SGI11*c* (*n* = 1), SGI11*d* (*n* = 1) and SGI11*e* (*n* = 2; Fig. 2).

Usually, SGI11 genomic islands contained antimicrobial resistance genes (*bla*_TEM-1_, *catA1*, *strA*, *strB*, *sul1*, *sul2* and *dfrA7*), mercury resistance genes (*merE*, *merD*, *merA*, *merC*, *merP*, *merT* and *merR*; Fig. 2a) and the *qacEΔ1*gene that encodes ethidium-bromide resistance protein, a member of the small multidrug resistance (SMR) family [31]. SGI11 was previously found to interrupt the *nlpC* or *yidA* gene (Chiou et al. [15]. All archetypical SGI11 in our Bangladeshi isolates disrupted the *yidA* gene, which encodes a sugar phosphate phosphatase. One isolate that contained the archetypical SGI11 sequence had inversion of the segment containing the *strB*, *strA* and *sul2* genes (Fig. 2a, red dotted bridge); this inversion was also observed in SGI11*d* (Fig. 2d). Similarly to archetypical SGI11, SGI11*b, c,* and *d* also disrupted the *yidA* gene, but contained deletions of 7857 bp (region encoding *bla*_TEM-1_, *strA*, *strB* and *sul2*), 1317 bp (region encoding *catA1*), or 9959 bp (region encoding *catA1*, *sul1*, *dfrA7* and *qacEΔ1*), respectively (Fig. 2b-d). SGI11*e* was located between the *cyaY* and *cyaA* genes, missing a 5651 bp region (encoding *strB*, *strA* and *sul2*) and the direction of the AMR genes and IS elements were reversed (Fig. 2e). None of the SGI11 variants interrupted the *nlpC* gene, which has been described previously by Chiou et al. [15] as one of the probable insertion sites for the island.

### Plasmids

Five different types of plasmids were detected and assembled, and ranged in size from 43,427–218,627 bp (Table 1). In total, 50 of the 73 isolates harbored plasmids: 49 isolates harbored just one type of plasmid and isolate 311189_217186 harbored two plasmids, matched (by homology) with NC_003384 and NC_003385, known as pHCM1 and pHCM2 respectively [32]; see Table 1.

**Table 1:** Plasmids and genomic islands detected in the 73 *S.* Typhi isolates. The resistance genes present in these elements are listed

	Name	Size range (bp) or location	Number	Resistance genes
Plasmids	pHCM1	214,596 - 218,627	20	*bla*_*TEM-1*_ *; catA1; strA; strB; sul1; sul2; dfrA7; qacEΔ1*
	pHCM2	106,706 - 106,706	21	–
	pK43	43,427	1	–
	pPRJEB21992	88,544	1	*bla*_*TEM-1*_ *; bla*_*CTX-M-15*_
	pK91	91,848 - 93,445	7	*bla*_*TEM-1*_ *; sul2; qnrS1; tetA; tetR*
Genomic Islands	SGI11	*yidA*	8	*bla*_*TEM-1*_ *; catA1; strA; strB; sul1; sul2; dfrA7; qacEΔ1*
	SGI11*b*	*yidA*	9	*catA1; sul1; dfrA7; qacEΔ1*
	SGI11*c*	*yidA*	1	*bla*_*TEM-1*_ *; strA; strB; sul1; sul2; dfrA7; qacEΔ1*
	SGI11*d*	*yidA*	1	*bla*_*TEM-1*_ *; strA; strB; sul2*
	SGI11*e*	*cyaY-cyaA*	2	*bla*_*TEM-1*_ *; catA1; sul1; dfrA7; qacEΔ1*

Twenty pHCM1-like plasmids were assembled, and ranged in size from 214,596–218,627 bp. All pHCM1-like plasmids harbored similar resistance genes as SGI11 (Fig. 2a). We also assembled 21 pHCM2-like plasmids; 17 were 106,706 bp and four were 106,705 bp, ~ 200 bp longer than the reference pHCM2 (NC_003385) plasmid sequence of *S.* Typhi CT18. However, the gene content of the short and long pHCM2 plasmids were the same as the reference [32].

**Fig. 3:**
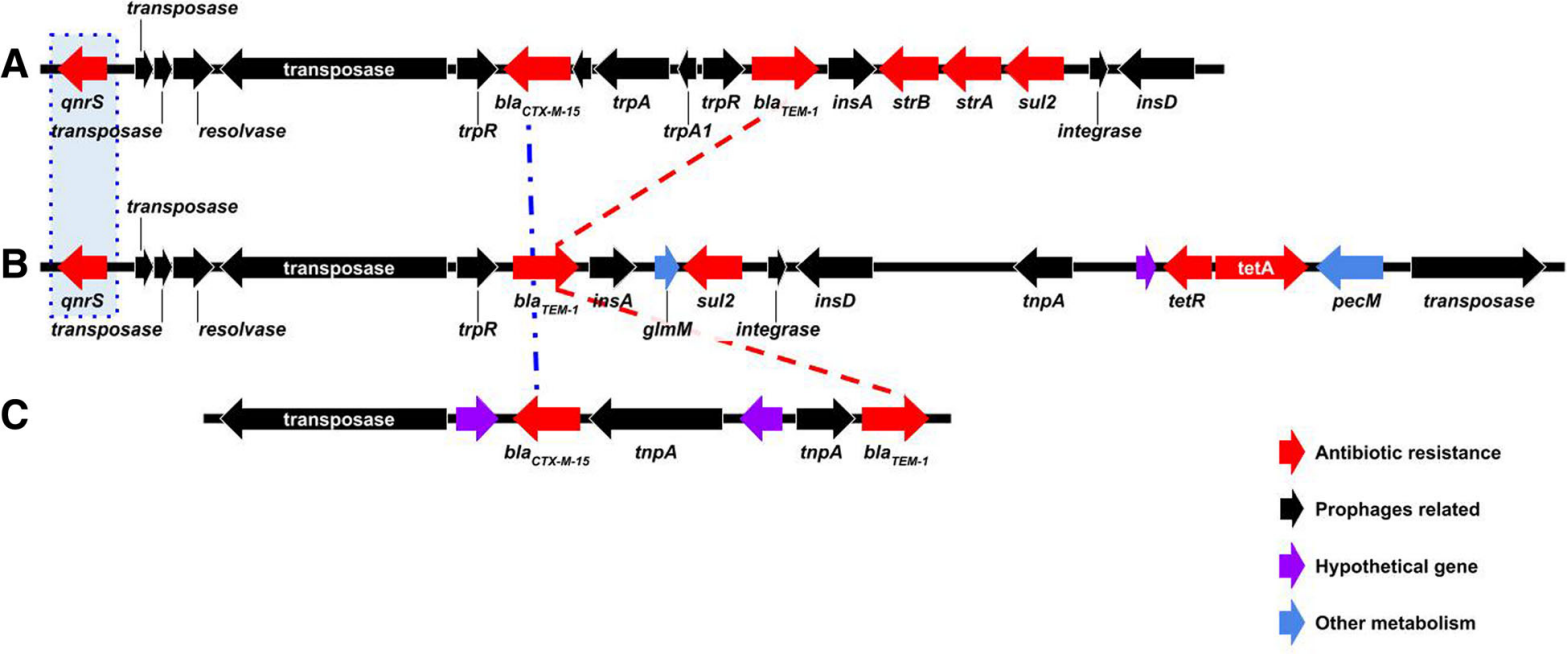
Comparison of the resistance-gene containing regions of the plasmids **a** p60006, **b** pK91 and **c** pPRJEB21992. The dashed blue box indicates the *qnrS* gene common to p60006 and pK91. The dashed blue line indicates the *bla*_CTX-M-15_ gene common to p60006 and pPRJEB21992. The red dashed line indicates *bla*_TEM-1_ is common to all three plasmids

Another plasmid, which we named pK91, was present in seven isolates. This plasmid ranged in size from 91,848–93,445 bp, harbored the *qnrS1*, *bla*_TEM-1_, *sul2*, *tetR*, and *tetA* resistance genes, and shared 66% query coverage (at 99% identity in the aligned portions) with a plasmid from *E. coli* (CP026578). Our single ceftriaxone-resistant isolate contained an 88,544 bp plasmid, previously described as pPRJEB21992, that harbored *bla*_TEM-1_ and *bla*_CTX-M-15_ [11]. Both pK91 and pPRJEB21992 contained a compendium of type IV secretion system genes. Recently, Klemm et al. [10] described a promiscuous plasmid, p60006, that confers resistance to fluoroquinolones and third-generation cephalosporins. This plasmid harbored the *qnrS1*, *bla*_TEM-1_ and *sul2* genes (similarly to pK91) and the *bla*_CTX-M-15_ and *bla*_TEM-1_genes (similarly to pPRJEB21992). Figure 3a, b and c shows a comparison of the regions carrying resistance genes in p60006, pK91 and pPRJEB21992, respectively. One isolate (ID: 343077_281186) contained a 43,427 bp plasmid, which we named pK43, with high similarity to the 38 Kbp pSTY1 (CP009103) plasmid from *Salmonella* Typhimurium strain ATCC 13311. This plasmid lacks resistance genes, but encodes genes for pili formation and conjugation.

### SGI11, plasmids and comparison with genotypes and MLST

**Fig. 4:**
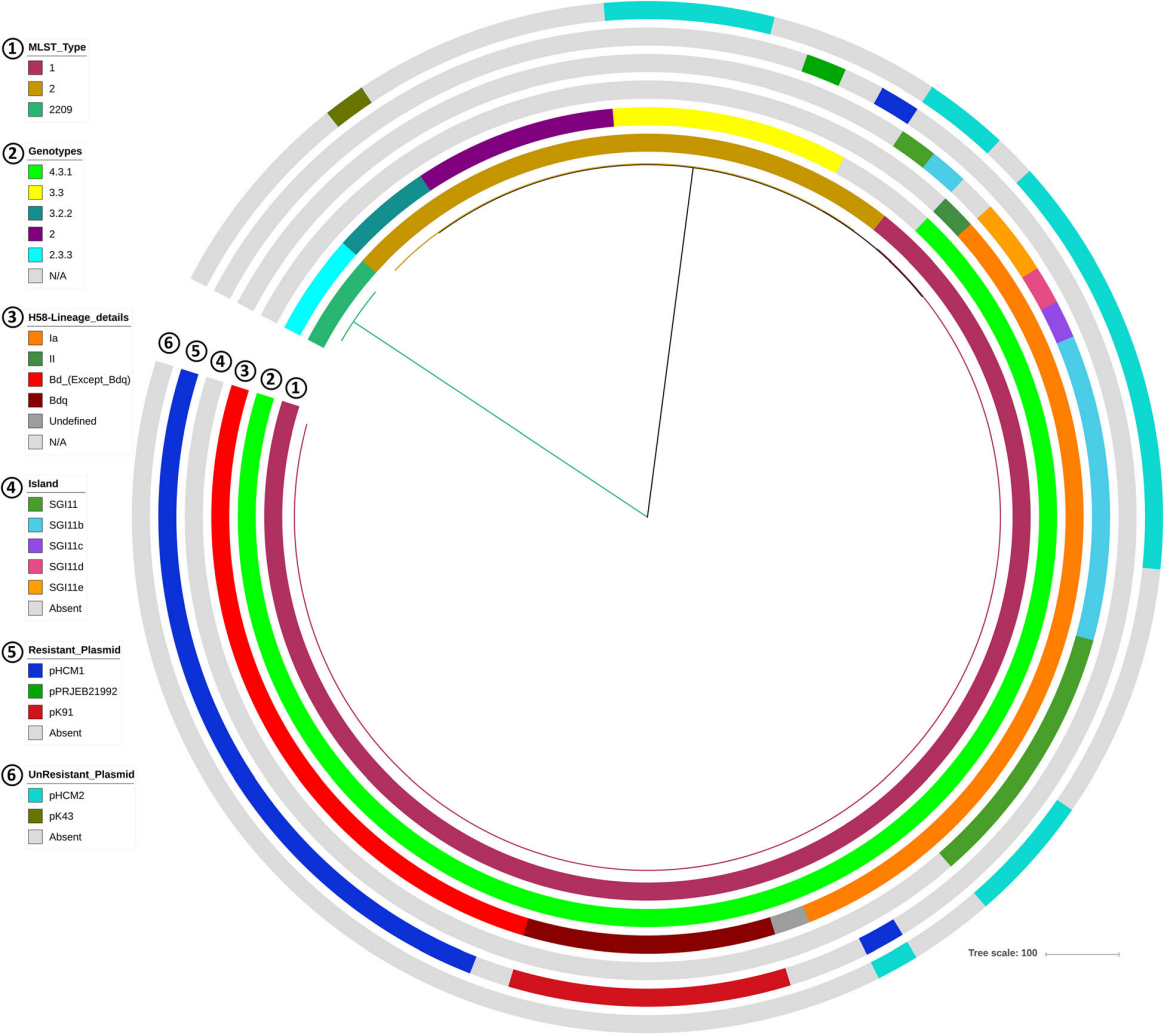
Comparison of 73 isolates from Bangladesh in a MLST-derived UPGMA tree. The tree is colored by MLST type. Different data points, including genotype, H58-lineage details, presence of different variants of SGI11, resistant_plasmids and unresistant_plasmid (which are not involved with AMR) are indicated in different circles around the tree (by colors)

Comparison of the SNP-based genotyping data with the presence of plasmids and SGI11 revealed few remarkable associations. Except for the three isolates (3/73) with undetermined genotype data, all isolates that carried pK91 plasmids (*n* = 7), pHCM1 plasmids (*n* = 20) or any variant of SGI11 (*n* = 21) were from genotype 4.3.1 (Haplotype 58, H58; Additional file 2: Table S3). Moreover, all isolates with SGI11 variants (19/70) belonged to H58-lineage Ia. No isolates from other H58 lineages or other genotypes contained SGI11 (Fig. 4, Additional file 2: Table S3).

In contrast, only one lineage Ia isolate had the pHCM1 plasmid. All other isolates with this plasmid (18/70) belonged to the newly described H58-lineage Bd; only one strain from this lineage did not contain the pHCM1 plasmid. However, none of these isolates were from the Bd sublineage *Bdq*. In contrast, all isolates from the *Bdq* sublineage (*n* = 7) carried a pK91 plasmid (Fig. 4, Additional file 2: Table S3).

All isolates carrying pHCM2 had either genotype 3.3 (*n* = 4) or 4.3.1 (*n* = 14). All isolates with the 4.3.1 genotype were from lineage Ia (*n* = 14). Only genotype 3.2.2 isolates carried the pK43 plasmid (Additional file 2: Table S3).

ST1 was the dominant MLST type among our isolates (*n* = 52), followed by ST2 (*n* = 18) and ST2209 (*n* = 3). Forty-six of the 52 ST1 isolates had either a resistance plasmid (pHCM1 or pK91) or a variant of SGI11. In contrast, only one of the 18 ST2 isolates carried pHCM1; no ST2 isolates carried pK91 and only one ST2 isolate had SGI11. None of the ST2209-type isolates had either a plasmid or SGI11.

The ceftriaxone-resistant isolate with the pPRJEB21992 plasmid had the 3.3 genotype and ST2 type.

### Resistance profiles

Our 73 isolates were classified into 12 resistance profiles (Table 2), of which two were multidrug resistant (MDR) and 10 were non-MDR (nMDR). Thirty-six of the 37 isolates with ampicillin resistance contained plasmids and/or SGI11 variants, namely pHCM1 (*n* = 20 isolates), pK91 (*n* = 6), pPRJEB21992 (*n* = 1), SGI11 (*n* = 6), SGI11*c* (*n* = 1), SGI11*d* (*n* = 1) and SGI11*e* (*n* = 2; Table 3, Additional file 2: Table S3). Two isolates with SGI11 and one with pK91 were susceptible to ampicillin, despite carrying *bla*_TEM-1_. Isolates with SGI11*b*, pK43, and pHCM2 were *bla*_TEM-1_*-*free and susceptible to ampicillin, with one exception: one isolate harboring SGI11*b* without *bla*_TEM-1_ was ampicillin-resistant.

**Table 2:** Resistance profiles of the *S.* Typhi isolates. (R) resistant; (S) susceptible; amp, ampicillin; sxt, cotrimoxazole; chl, chloramphenicol; cip, ciprofloxacin; cro, ceftriaxone

Number of Isolates	Resistance Profile	Plasmids/Genomic Islands present
26	amp-R, sxt-R, chl-R, cip-R, cro-S	pHCM1 (20); SGI11 (5)
15	amp-S, sxt-S, chl-S, cip-R, cro-S	SGI11(1); pK91 (1)
10	amp-S, sxt-S, chl-S, cip-S, cro-S	–
7	amp-R, sxt-S, chl-S, cip-R, cro-S	SGI11*d* (1); pK91 (6)
6	amp-S, sxt-R, chl-R, cip-R, cro-S	SGI11 (1); SGI11*b* (5)
2	amp-S, sxt-S, chl-R, cip-R, cro-S	SGI11*b* (2)
1	amp-R, sxt-R, chl-R, cip-S, cro-S	SGI11 (1)
1	amp-R, sxt-R, chl-S, cip-R, cro-S	SGI11*c* (1)
2	amp-R, sxt-S, chl-R, cip-R, cro-S	SGI11*e* (2)
1	amp-R, sxt-S, chl-S, cip-S, cro-R	pPRJEB21992 (1)
1	amp-S, sxt-R, chl-R, cip-S, cro-S	SGI11*b* (1)
1	amp-S, sxt-S, chl-R, cip-S, cro-S	SGI11*b* (1)

**Table 3:** Summary of resistance to each antibiotic tested. The genes associated with a given resistance profile, as well as the number of susceptible (S) or resistant (R) isolates, are shown. For ciprofloxacin resistance, we also show mutations on *gyrA/B* and *parC/E* genes

	# resistant isolates	# susceptible isolates	Associated resistance gene (number of genes)	gene:mutation:number of isolates with mutations
Ampicillin	38	35	*bla-tem-1* (40)	*Not applicable*
Ceftriaxone	1	72	*bla-ctx-m-15* (1)	*Not applicable*
Ciprofloxacin	59	14	*qnrS1* (7)	*gyrA:S83Y:29*
				*gyrA:S83F:28*
				*gyrA:D87G:2*
				*gyrA:D87N:3*
				*gyrA:N529S:6*
				*gyrA*:D538N:52
				*gyrB:S464F:9*
				*parC:S80I:1*
				*parC:S80E:1*
				*parC:E84K:2*
				*parE:S339 L:1*
				*parE:A364V:7*
				*parE:L416F:1*
Chloramphenicol	39	34	*catA1* (39)	*Not applicable*
Cotrimoxazole	35	38	*sul1* (39); *sul2* (36); *dfrA7* (40)	*Not applicable*

Only one of the 73 isolates was resistant to ceftriaxone (Table 3, Additional file 2: Table S3). This isolate was also resistant to ampicillin, and harbors the pPRJEB21992 plasmid that encodes both bla_TEM-1_ and *bla*_CTX-M-15_. In terms of ciprofloxacin resistance, we identified multiple mutations in the *gyrA* and *parC* genes, including S83F, S83Y, D87G, and D87N in *gyrA*; S80I, S80R, and E84K in *parC* (Table 3). A quinolone resistance (*qnr*) gene, *qnrS1* was detected in seven isolates and carried by a plasmid (herein called pK91) which also had a *bla*_TEM-1_ gene [33] (Table 3). Both *gyrA* and *parC* mutations, and the *qnr* genes were associated with resistance to ciprofloxacin (Additional file 2: Table S3). Other detected mutations in the non-QRDR regions include N529S, D538N of *gyrA*, S464F of *gyrB*, and S339 L, A364V and L416F of *parE* (Table 3).

The *catA1* gene was present in pHCM1, SGI11 and the SGI11*b* and *e* variants. Only one isolate with *catA1* showed susceptibility to chloramphenicol. Another isolate was phenotypically resistant to chloramphenicol, but did not contain a plasmid or SGI11 carrying the *catA1* gene.

Thirty-five isolates were resistant to cotrimoxazole. Of these, 27 had the three aforementioned *dfrA1*, *sul1*, and *sul2* genes, six had the *dfrA1* and *sul1* genes, and one isolate only had the *dfrA1* gene (Table 3, Additional file 2: Table S3). One cotrimoxazole-resistant isolate did not harbor *dfrA1*, *sul1* or *sul2* genes. Thirty-eight isolates were classified as being susceptible to cotrimoxazole, 24 of these isolates did not have any of the *dfrA1*, *sul1* or *sul2* genes. Eight cotrimoxazole-susceptible isolates had a *sul2* gene, five had only *dfrA1* and *sul1* genes, and one isolate had all three genes.

## Discussion

In agreement with previous findings [34], all 73 *S.* Typhi genomes in this study were highly conserved, as confirmed by the ANI values (> 98%; Additional file 1: Table S2) and whole genome alignments (Additional file 3: Figure S1). However, the presence or absence of SGI11-like elements led to sequence differences and variation in genome size.

The core genome analysis also indicated high genomic conservation between our 73 isolates, with 3944 genes in the *S.* Typhi core genome (60% of the pan-genome). In contrast, a previous study of only six isolates identified 4131 core genome genes [35]. The limited geographical origin (Bangladesh) of our isolates could explain the low number of genes in the core genome. Conversely, the higher number of isolates in this study (73 vs. 6 in Baddam et al., 2015) may explain the differences in the pan-genome content (6602 vs. 5426 genes). In contrast, the *Salmonella enterica* pan-genome has a higher gene number but smaller core genome than the *S.* Typhi isolates in this study. This difference is to be expected, as the *Salmonella enterica* dataset included multiple serovars [36]. Interestingly, our *S.* Typhi pan-genome contains a higher number of genes than *S.* Paratyphi-A (4670; 41 isolates) and *S.* Enteritidis (4750; 159 isolates), but a smaller number of genes than *S.* Typhimurium (7603; 47 isolates; [37]. These findings corroborate the hypothesis that the pan-genome size does not reflect the host range or ability to colonize multiple hosts [37, 38].

However, the accessory genome or serovar-specific core genome could be responsible for such host variety (Seif et al., 2018). Considering the host restriction of *S.* Typhi, the same rationale could explain why 93% of the average gene content of our isolates was present in the core genome. In contrast, serovars like *S.* Typhimurium with a variable host range have 75% of its average gene content in its core genome (3475/ 4661) [37]. Variation within a serovar should be reflected in the dispensable genome, whereas the unique genome should explain strain-specific characteristics. Our *S.* Typhi dispensable genome showed enrichment of COG classes X (*n* = 48), L (*n* = 38), K (*n* = 33), S (*n* = 23; function unknown), C (*n* = 22) and E (*n* = 21). Class X refers to genes related to prophages and transposons; we found ~ 50% of class X genes in the dispensable genome were located in the prophage regions of the *S.* Typhi genome. Moreover, ~ 40% of class L (DNA repair) genes in the dispensable genome were located in prophage regions. Prophage-like elements in *Salmonella* serovars can play a role in recombination, contribute to virulence in the host and carry specific virulence-associated genes such as *sopE*. In the case of *S.* Typhi, these regions could even cause more subtle intra-serovar variation (Boyd and Brüssow 2002; Thomson et al. 2004). These prophage regions have also been reported to contribute to mechanisms of DNA repair, possibly as a part of the bacterial SOS regulatory system (Balbontín et al. 2006). Our core *S.* Typhi genome lacked a number of COG classes, including Y (genes related to nuclear structure), B (*Chromatin structure and dynamics*) and Z (*Cytoskeleton*). This result could be an artifact of COG class annotation, as the current version of the COG database does not include in their classes bacterial genes related to chromatin-like organization or cytoskeletal formation, even though there are only but a few genes on those related functions for bacteria. Also there may be a lack of annotation of genes related to those classes.

Remarkably, our analysis of 73 *S.* Typhi genomes suggested an open pan-genome, in contrast to previous studies that reported *Salmonella enterica* had a closed pan-genome [36, 39]. However, these previous studies were mainly performed on the *Salmonella* genus, not specifically the Typhi serovar. Moreover, the low numbers of *S.* Typhi isolates in these studies could lead to discrepancies in the pan-genome results [36, 39]. An open pan-genome usually indicates bacterial species that can colonize multiple environments and exchange genetic material in multiple ways. Other more conserved species that tend to live in isolated niches with limited access to the microbial gene pool or that have a lower capacity to acquire foreign genes usually show a closed pan-genome [40]. Considering its human host-restriction, *S.* Typhi should have a closed pan-genome. However, typhoid fever is endemic in many highly-populated areas of the world, where the bacteria are transmitted through contaminated food and water. Such transmission mediums contains vast bacterial community, acting as a bacterial gene pool, which may reflect in an open pangenome [36, 39]. Moreover, prophage regions of the genome may work as hotspots for the acquisition of new genes in those regions [41]. The core/pan-genome ratio, 0.6 (3944/ 6602) in our study also points to open pangenome, as discussed previously by Rouli et al. 2015 [41]. In Addition, the number of pseudogenes in our *S.* Typhi isolates was higher; median 177 (4%), similar to ~ 200 in *S*. Typhi CT18 reference genome, compared to *S.* Typhimurium (0.9%) or, *Escherichia coli* K12 (0.7%). A high number of pseudogenes in bacterial species could be associated with its host restriction, as it has been observed in other bacteria [34]. Certain genes required for a broad range of hosts may become pseudogenes, once bacteria get adapted to only one preferred host [34]. Host-specificity of *S.* Typhi is mostly due to the strong selectivity of typhoid toxin for Neu5Ac- terminated glycans over Neu5Gc-terminated ones and the absence of an operational Rab32-dependent host defense pathway in human. Neu5Ac- terminated glycans are predominantly expressed in human cells, while the Neu5Gc- terminated ones are dominant in other mammals [42–44]. It could be the reason why *gtgE* (a cysteine protease) and *sopD2* (Type III secretion system effector protein) genes became pseudogenes or missing in *S.* Typhi genome, as reported earlier [32, 42]. Both the genes are present in other *Salmonella* (e.g. Typhimurium) to protect the bacteria from Rab32-dependent host defense pathway in other mammalian hosts (e.g. Chimpanzees) [32, 42].

Despite high sequence conservation, we observed some differences in the resistance gene contents of our isolates, and these differences were reflected in the phenotypic resistance profiles (Table 2). This variation in resistance can be attributed to the mutations occurring on antibiotic target genes and the presence of acquired resistance genes carried on plasmids or SGI11-like islands. Different variants of SGI11, with different gene contents or orientations and locations in the chromosome, were observed among our isolates. Four of the five SGI11 variants interrupted the *yidA* gene of the *S.* Typhi genome, while SGI11e was located between the *cyaY* and *cyaA* genes (Table 1); both locations have previously been described [15, 16, 18].

The presence of the archetypal SGI11 sequence, SGI11*b, d or e*, or pHCM1 conferred chloramphenicol resistance, as these elements harbor the *catA1* gene; the *catA1* gene was missing from SGI11*c.* Only two isolates did not exhibit the corresponding resistance or susceptibility phenotypes based on the presence or absence of *catA1*. However, even if the *catA1* gene is absent, other mechanisms such target gene mutations or the presence of an efflux pump can confer resistance [45]. In contrast, a decrease in the concentration of acetyl-CoA can inhibit the activity of *catA1* and lead to a susceptibility phenotype, even in the presence of the *catA1* gene [46].

Among the isolates exhibiting an ampicillin-resistant phenotype, the *bla*_TEM-1_ beta-lactamase gene was present in all SGI11 variants (except variant *b*) and the pHCM1, pK91 and pPRJEB21992 plasmids (Figs. 2 and 3). However, three isolates were susceptible to ampicillin despite harboring the *bla*_TEM-1_ resistance gene; this could be related to altered transcriptional control due to a weak *bla* gene promoter [47]. However, analyses of the promoter regions did not reveal any variation (data not shown). Ampicillin resistance in the absence of a *bla*_TEM-1_ gene was also observed for one isolate, which may indicate the involvement of other resistance mechanisms like overexpression of efflux pump genes [48–50].

Unlike ampicillin and chloramphenicol, cotrimoxazole is a drug combination of trimethoprim and sulfamethoxazole, which exert a synergistic bacteriostatic effect. One mechanism of cotrimoxazole resistance involves the acquisition of folate-biosynthesis pathway genes that are resistant to the bacteriostatic effect [51, 52]. These resistance genes can be carried by plasmids or integrons, and the combined presence of a resistant dihydropteroate synthase gene (*sul1* or *2*) and dihydrofolate reductase (*dfr*) can confer resistance to cotrimoxazole. Indeed, most of our resistant isolates contained the *dfrA7*, *sul1* and *sul2* genes. Other isolates had either *dfrA7* and *sul1,* or only *dfrA7*. The *sul2* gene does not confer a resistance phenotype on its own. In contrast, six of our cotrimoxazole-susceptible isolates had a *dfrA7* gene with a *sul1* and/or *sul2* gene. However, similar discrepancies were also reported from other studies who compared whole genome sequence (WGS) with antimicrobial susceptibility data [53–56].

Only one of the 73 isolates exhibited a resistance phenotype to ceftriaxone, which could be explained by the presence of a *bla*_CTX-M-15_ gene on a pPRJEB21992 plasmid. A highly ceftriaxone-resistant *S.* Typhi was previously reported in Bangladesh in 1999 [57], but this isolate was not subjected to molecular characterization. Djeghout et al. [11] described the first assembled plasmid harboring *bla*_CTX-M-15_, pPRJEB21992, from a Bangladeshi strain isolated in 2000. We studied the same strain to compare it with other resistance plasmids we found in this study. Another plasmid, p60006, which harbors the same gene for ceftriaxone resistance was reported in a *S*. Typhi strain that caused an outbreak in Pakistan during 2016 and 2017 [10]. Both plasmids, pPRJEB21992 and p60006, may be the same type, but have different gene contents. Moreover, these plasmids possibly have different evolutionary origins or took different patterns of divergence, as whole-genome SNP (wgSNP) analyses revealed genotypic and phylogenetic differences between the isolates, including differences in the *bla*_CTX-M-15_ gene sequence (99% identity and 92% coverage); [58]. Considering the origin of both of these plasmids as independent events, the chance of strains carrying any of these plasmids spreading is low, but cannot be ruled-out as both plasmids are extra-chromosomal elements. A significant increase of AMR may occur if strains carrying either of these two plasmids spread outside of their current geographical origin.

Unlike other antimicrobial agents, resistance to ciprofloxacin (cip) was common among our isolates and was associated with mutations in the *gyrA*/*B* and *parC*/*E* genes, which encode the DNA gyrase and topoisomerase IV enzymes, respectively. Indeed, 55 of the 59 cip-resistant isolates contained the S83F and S83Y mutations in the *gyrA* gene. Another *gyrA* mutation, D538N was also common (52/73 isolates), but is not located in the QRDR region of the gene and should not influence susceptibility to cip [59]. Moreover, this *gyrA*-D538N mutation and two other mutations, *gyrA*-N529S and *parE*-A364V were associated with the different genotypes of the *S.* Typhi isolates but were not involved in cip resistance [58]. Resistance can also be conferred by the *qnr* genes [60]. Remarkably, the seven isolates with pK91 plasmids containing the *qnrS1* gene had high cip MICs (> 4.0 μg/mL, Additional file 2: Table S3). These isolates also contained the *gyrA-*S83Y mutation, but did not have other mutations in the *gyrB*, *parC* or *parE* genes. The wgSNP analysis identified these isolates are part of a highly cip-resistant local H58-sublineage, Bdq [58]. *S.* Typhi isolates from a Pakistani outbreak also contained *qnr* genes in the p60006 plasmid and were highly resistant to ciprofloxacin [10].

The gene contents of the p60006, pK91 and pPRJEB21992 plasmids were noticeably different (Fig. 3). However, the presence of a type IV secretion system and common IS elements in these plasmids suggest a common ancestor, and then independent patterns of divergence. The pHCM2 and pK43 plasmids had no association with resistance or other metadata (Additional file 4: Table S1 and Additional file 2: Table S3).

Surprisingly, the presence of the pHCM1 and pK91 plasmids and SGI11 islands were associated with the *S.* Typhi genotype and H58 lineage. With the exception of pPRJEB21992, all AMR-related plasmids and SGI11 were detected in isolates with genotype 4.3.1 (Haplotype 58, H58). The seven isolates with high cip MICs that harbored the pK91 plasmid were from the newly reported H58 sublineage, Bdq (Tanmoy et al., 2018). The isolates carrying pHCM1 plasmids, which confer MDR and cip resistance, were from the local Bd lineage (but not the Bdq sublineage), while isolates with SGI11 were from lineage Ia (Additional file 2: Table S3). Thus, the local *S.* Typhi lineage, Bd, appears to be less prone to chromosomal integration of the MDR locus than the globally widespread lineage Ia. The presence of pHCM1 and/or pK91 plasmids in lineage Bd isolates could also suggest the unaltered fitness of the lineage. Cip resistance conferred by *gyrA/B* and *parC/E* mutations did not provide any fitness advantage either, as they cannot offer any as previously reported [61]. However, the presence of *gyrA/B* and *parC/E* mutations in 72 of our 73 isolates could indicate strong selective pressure on the genome from the overuse of antimicrobials [34].

The effect of such anthropological selective pressure could be particularly evident in Bangladesh and other South Asian countries. Self-medication and over-the-counter sale of antibiotics, especially ciprofloxacin, is prevalent in this region; ciprofloxacin has been one of the preferred treatments for enteric or diarrheal diseases since the 2000s [62, 63]. The high concentrations of this drug in meat products (chicken and livestock) can also contribute to cip resistance in the environment, leading to increased selective pressure [64, 65]. This strong selective pressure could have played a crucial role to limit the spread of lineage Bd to specific geographic regions. In contrast, lineage Ia may represent the major evolutionary event of integration of the MDR locus into the chromosome to maintain the MDR phenotype, as well as a gain of fitness advantages [16].

Besides revealing these characteristics of *S.* Typhi, we obtained the complete chromosome sequences for 73 isolates, which substantially increases the number of complete (closed) chromosome sequences for this serovar available in the NCBI (only 46 sequences were available until now). However, the isolates in this study were only collected from pediatric patients as the disease is most common among school-aged children, but typhoid can occur at any age. Thus, only studying isolates from pediatric cases may not provide a complete picture of *S.* Typhi in Bangladesh. Moreover, the number of isolates in our study (*n* = 73) may be too low to detect all genetic changes that have occurred over the 15 years between 1999 and 2013, specifically chromosomal integration of the MDR locus. All of our isolates were from Bangladesh, which makes our pan-genome data relatively country-specific. However, as a tropical country where typhoid is endemic, our core and pan-genome data should reflect the scenario of a region where *S.* Typhi is endemic.

## Conclusions

We assembled and annotated complete chromosome and plasmid sequences for 73 *S.* Typhi isolates using only short-length Illumina reads. The isolates exhibited a highly conserved genome, with an open pan-genome. We report two new plasmids, pK43 with no link to resistance and pK91 that confers a high level of ciprofloxacin resistance. Multiple variants of SGI11 with different resistance genes were detected, and result in different resistance phenotypes. Plasmids carrying resistance genes were also present in many isolates with different phenotypes. The presence of SGI11 and plasmids encoding resistance genes (pHCM1 and pK91) were associated with two different H58 lineages, Ia and Bd, respectively. Shedding the plasmids and integration of the resistance genes into the genome (as islands) may have contributed to the fitness of the lineage Ia isolates; this could be one explanation for the wider geographical spread of this lineage, in comparison to the local lineage Bd that has remained restricted to Bangladesh. The results of this study should help us to better understand the multiple variations in the genomic elements that confer AMR in *S.* Typhi. Continuous surveillance of these elements could reveal other mechanisms by which AMR can spread in *S.* Typhi. However, preventive measures to minimize the spread of AMR should also be implemented, for example vaccination could be an effective tool to reduce the number of cases by preventing the overuse of antibiotics [66].

## Methods

### Bacterial strains and resistance profiles

All *Salmonella* Typhi isolates used in this study were collected by the Child Health Research Foundation (CHRF) from the blood of pediatric patients hospitalized at Dhaka Shishu (Children) Hospital (DSH) or pediatric outpatients treated at the Popular Diagnostic Centre (PDC) in Dhaka, Bangladesh. The CHRF team has been preserving *Salmonella* isolates since 1999 and currently maintains a biobank of over 3500 isolates.

Seventy three *S.* Typhi isolates were selected from the biobank based on their antimicrobial resistance phenotype (Table 2). We re-confirmed the identity of all strains using standard biochemical tests and agglutination tests with specific antisera for *Salmonella* species (Thermo Scientific, Waltham, MA, USA). Antimicrobial susceptibility to ampicillin (amp), cotrimoxazole (sxt) and chloramphenicol (chl) were determined using disk diffusion assays (Oxoid; Thermo Scientific). Broth-microdilution was employed to determine the minimum inhibitory concentrations (MIC) for ciprofloxacin (cip) and ceftriaxone (cro) (Sigma Aldrich, St. Louis, MO, USA). All zone diameters and MIC data were interpreted according to EUCAST v8, 2018 ([67], 2018). Metadata for all 73 isolates (sample, organism, year of isolation, setting, patient age [months] and sex) are presented in Additional file 4: Table S1.

### DNA sequencing, genome assembly, genome annotation and comparative genomics

Isolates were cultured on MacConkey agar (Oxoid, Thermo Scientific) overnight, checked for visible contamination (and re-plated if any contamination was observed), and all colonies were picked and suspended in water. QIAamp DNA Mini Kits (Qiagen, Hilden, Germany) were used to extract DNA from the suspensions on the same day. Whole genome sequencing (WGS) was performed using an Illumina-HiSeq 4000 platform, generating 2 × 150 bp paired-end reads with an average coverage of 121x, at The Oxford Genomics Centre of the Wellcome Trust Centre for Human Genetics, Oxford, UK.

Quality assessment of sequencing reads was conducted using FastQC [68]. Due to the high-quality scores of the reads and absence of adapter sequences, quality and read trimming were deemed unnecessary. Paired-end reads were first assembled using Newbler v3.0 [69]. More than 99.37% of assembled bases for all isolates had Q40 or more, as calculated using Newbler. We used JSpecies [70] to calculate the average nucleotide identity (ANI) with BLAST ([ANIb]; [71] and Mummer ([ANIm]; [72] for all isolates plus *Salmonella enterica subsp. enterica* serovar Typhi str. CT18 (accession NC_003198.1). ANIb/ANIm of our isolates in comparison to *S.* Typhi CT18 were higher than 99,85% which allowed scaffolds to be aligned against *S*. Typhi CT18 using cross_match [73] to create a layout of ordered and oriented scaffolds to be concatenated with gaps between scaffolds estimated from the alignment and filled with ‘N’. The layout of scaffolds was subjected to manual curation in order to 1) verify any missing scaffolds on the alignment; 2) confirm gap sizes; and 3) confirm the expected number of repeated scaffolds in agreement with their read coverage estimated by Newbler. This curated superscaffold was subjected to gap filling in two steps. In the first step, we used GapFiller v1.11 [74]. In step 2, remaining intra- and inter-scaffold gaps that were not closed by GapFiller v1.11 were locally assembled using Newbler v3.0. Reads present at both ends of a gap were selected and assembled. Contigs obtained this way and that spanned the gap and anchored on consensus sequences on both sides were added to the consensus, thus filling the gap. Step 2 was done manually to each remaining gap from step 1 and subjected to confirmation of each closed gap to avoid erroneous gap filling. Scaffolds that did not align to the chromosome of *S*. Typhi CT18 were aligned to the *nt* NCBI database in order to verify if they had plasmid origin. Those with plasmid origin were assembled using the approach described previously. Visual aid to the assembly process can be found in the Additional file 5: Figure S2. A similar approach was previously used to close a *Klebsiella* genomes [75]. The SABIA pipeline was used for automated annotation [76]. Assembled sequences were submitted to the NCBI (accession IDs are shown in Additional file 4: Table S1).

During the superscaffold formation step, the presence of a *Salmonella* genomic island (SGI) was noticed in 21 strains, due to careful manual curation of the superscaffold formation step. This island carries several resistance genes and is not present in the reference *S*. Typhi genome. Based on sequence similarity and gene content, the SGI was identified as *Salmonella* genomic island 11 ([SGI11] accession number KM023773; [15]. According to Chiou et al. [15], SGI11 can be located at two chromosomal positions, interrupting the *yidA* gene or *nlpC* gene. Contigs were aligned against the SGI11, *nlpC* and *yidA* sequences to determine the presence of the island and its position of insertion. For strains in which there was evidence of a SGI11 element but neither *yidA* nor *nlpC* were interrupted, the extremities of the contigs harboring the segments of the island were locally assembled and the gene neighborhood was determined after annotation.

To observe conserved synteny blocks, we aligned all isolates and *S.* Typhi CT18 using Progressive Mauve [77]. We used RPS-BLAST, a variant of PSI-BLAST [78] to identify proteins with homology to COG protein profiles in the NCBI database at an e-value of 0.001. The best hit was selected and COG cluster classification was transferred to the protein query. Pan and core genome analysis was performed using PGAP v1.2.1 [79]. Coding sequences, protein sequences and COG classification for all isolates were used as input, along with an e-value of 1e-10 at 70% identity and similarity. All protein sequences were aligned against each other using blastall and the resulting output was imported into MCL [80], a part of PGAP, to cluster the genes. After clustering, PGAP computes the pan and core genomes by strain combination from 1 to *n* strains, where *n* denotes the maximum number of strains (*n* = 73 in this study). Heap’s law and an exponential law were employed to fit the pan and core genomes, respectively [79]. PanGP was used to plot the pan and core genome curves [81].

After assembly and annotation of the chromosomes and plasmids, AMR genes on the SGI11 and plasmids were manually annotated. We also used Abricate [82] to corroborate the AMR genes using the following databases: Resfinder, ARG-ANNOT, CARD, NCBI Bacterial Antimicrobial Resistance Reference Gene Database, EcOH, PlasmidFinder, VFDB and Ecoli_VF. We verified prophage regions on the chromosome and plasmids with PHASTER [83] to verify the presence of some classes of dispensable genome genes on these elements.

We previously obtained genotype data for 70 of the 73 isolates in this study [58]. The multilocus sequence typing (MLST) data for the isolates was determined using Enterobase [84]. The MLST data was used to build a UPGMA phylogenetic tree. The Euclidean distance matrix and UPGMA tree were computed using the *dist* and *hclust* functions, respectively, of the R stats package. The tree was displayed and annotated using the online version of iTOL v4 [85].

## Additional files

Additional file 1: **Table S2.** Sheet 1. General information about the isolates assembled. Sheet 2. Average Nucleotide Identity calculated by BLAST. Sheet 3. Average Nucleotide Identity calculated by MUMMER. (XLSX 71 kb)

Additional file 2: **Table S3.** Ampicilin, ciprofloxacin, chloramphenicol, ceftriaxone, and cotrimoxazole resistance. (XLSX 50 kb)

Additional file 3: **Figure S1.** Isolate chromosomes were aligned with ProgressiveMauve. Conserved sequence block can be observed as they are marked with the same color. (JPEG 4269 kb)

Additional file 4: **Table S1.** Sheet NCBI ID: Accession numbers for sequences deposited on NCBI. Sheet Metadata: metadata for the isolates studied. (XLSX 15 kb)

Additional file 5: **Figure S2.** Schematic summary of the assembly and gap filling process. (JPG 256 kb)

## Data Availability

Sequence and annotation data that support the findings of this study have been deposited in GenBank with the accession codes being listed in Additional file 4: Table S1.

## Abbreviations

amp: Ampicillin
AMR: Antimicrobial resistance
ANI: Average nucleotide identity
ANIb: Average nucleotide identity with blast
ANIm: Average nucleotide identity with MUMMER
bp: Base pairs
chl: Chloramphenicol
CHRF: Child Health Research Foundation
cip: Ciprofloxacin
COG: Cluster of orthologous groups
cro: Ceftriaxone
DSH: Dhaka Shishu (children) Hospital
ESBL: Extended-spectrum beta-lactamase
GC: Guanine and cytosine
Kbp: Kilobase pairs
Mbp: Megabase pairs
MDR: Multidrug resistance
MIC: Minimum inhibitory concentration
MLST: Multilocus sequence typing
NCBI: National Center for Biotechnology Information
nMDR: Non-multidrug resistant
PDC: Popular Diagnostic Centre
PMQR: Plasmid-mediated quinolone resistance
Q40: Quality score 40
QRDR: Quinolone resistance determining region
-R: Resistant
-S: Susceptible
SGI: Salmonella genomic island
SMR: Small multidrug resistance
ST1: Sequence type 1
ST2: Sequence type 2
sxt: Cotrimoxazole
WGS: Whole genome sequence
wgSNP: Whole genome single nucleotide polymorphism
