# Supplementary figures and images for: Analysis of isolates from Bangladesh highlights multiple ways to carry resistance genes in Salmonella Typhi
Source: BMC Genomics. 2019 Jun 28;20:530. doi: 10.1186/s12864-019-5916-6 (PMC6599262; doi:10.1186/s12864-019-5916-6)

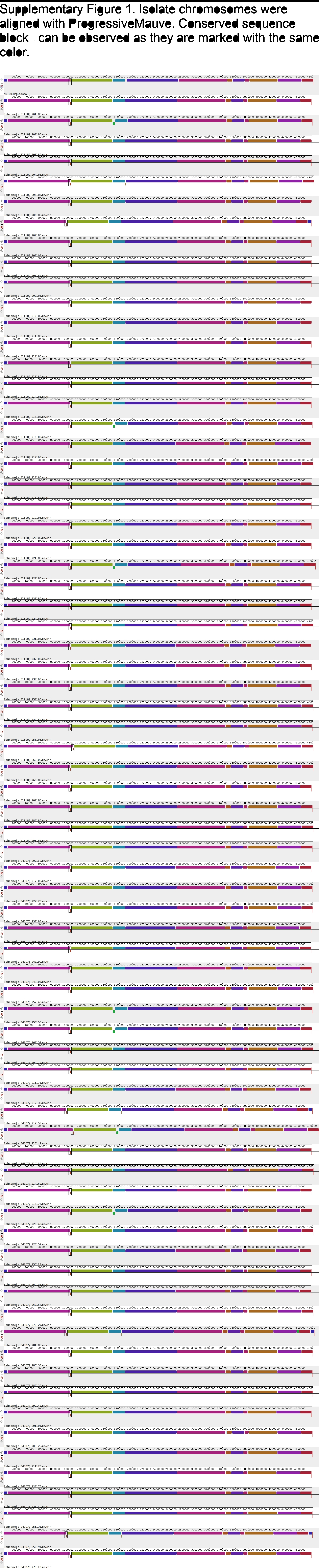

Supplement: Supplementary file 3 — Figure S1. Isolate chromosomes were aligned with ProgressiveMauve. Conserved sequence block can be observed as they are marked with the same color. (JPEG 4269 kb) [file 12864_2019_5916_MOESM3_ESM.jpeg]

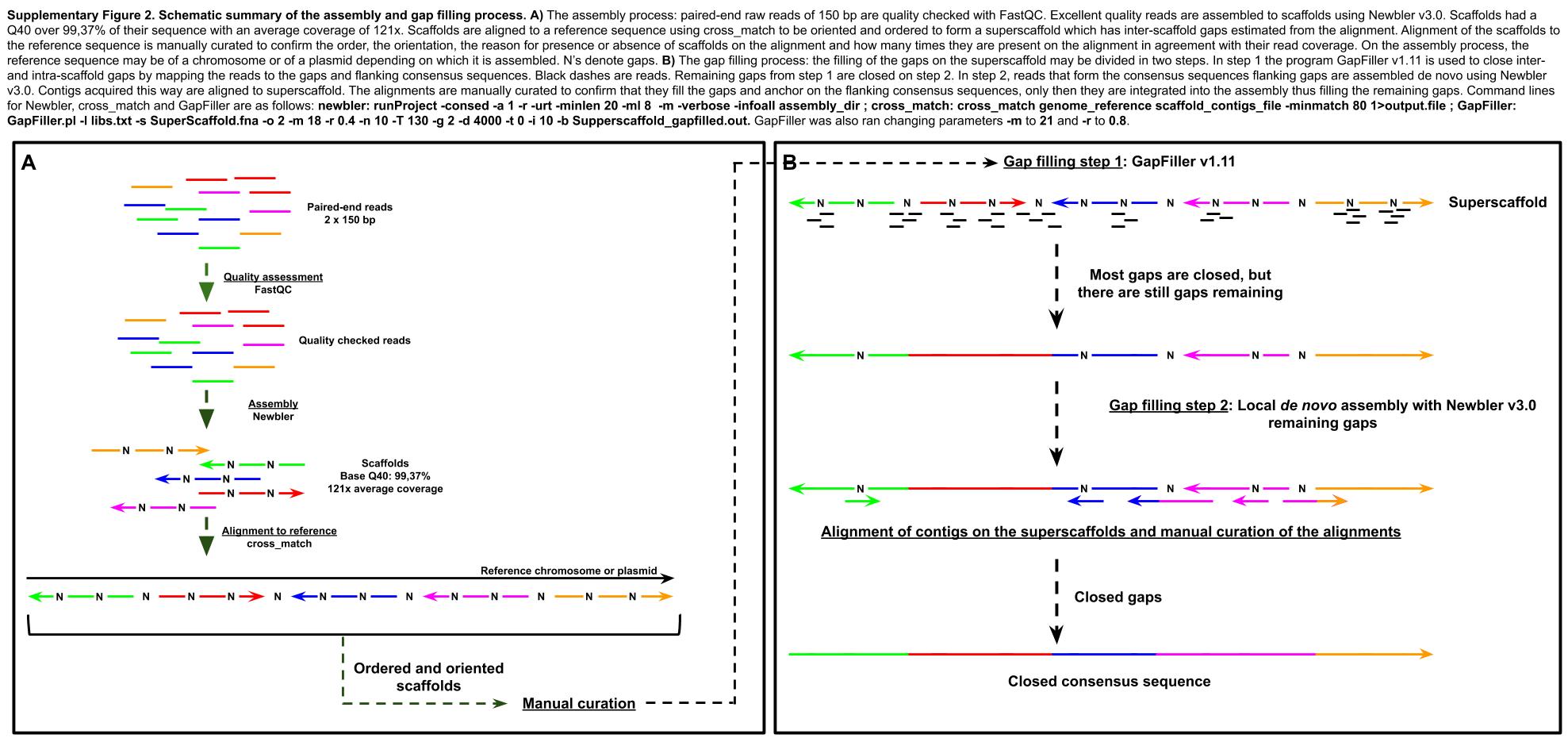

Supplement: Supplementary file 5 — Figure S2. Schematic summary of the assembly and gap filling process. (JPG 256 kb) [file 12864_2019_5916_MOESM5_ESM.jpg]
